# Zopfiellasins A–D, Two Pairs of Epimeric Cytochalasins from Kiwi-Associated Fungus *Zopfiella* sp. and Their Antibacterial Assessment

**DOI:** 10.3390/molecules26185611

**Published:** 2021-09-16

**Authors:** Jie-Yu Zhang, Juan He, Zheng-Hui Li, Tao Feng, Ji-Kai Liu

**Affiliations:** 1 School of Pharmaceutical Sciences, South-Central University for Nationalities, Wuhan 430074, China; zhangjieyu98@163.com (J.-Y.Z.); 2015048@mail.scuec.edu.cn (J.H.); 2015051@mail.scuec.edu.cn (Z.-H.L.); 2 National Demonstration Center for Experimental Ethnopharmacology Education, South-Central University for Nationalities, Wuhan 430074, China

**Keywords:** *Zopfiella* sp., cytochalasins, *Pseudomonas syringae* pv. *actinidiae*, antibacterial

## Abstract

In our continuous search for antibacterial agents against *Pseudomonas syringae* pv. *actinidiae* (Psa) from kiwi-associated fungi, two pairs of epimeric cytochalasins, zopfiellasins A–D (**1**–**4**), were characterized from the fungus *Zopfiella* sp. The structures were established on the basis of spectroscopic data analysis, while the absolute configurations were determined by single-crystal X-ray diffraction. Compounds **1** and **3** exhibited antibacterial activity against Psa with MIC values of 25 and 50 μg/mL, respectively. This is the first report of anti-Psa activity of cytochalasin derivatives.

## 1. Introduction

**Figure 1:**
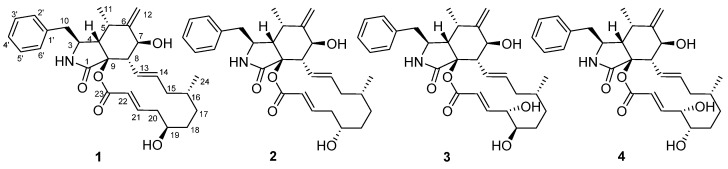
Chemical structures of compounds **1**–**4**.

Kiwi is a fruit produced by *Actinidia chinensis* and various artificially cultivated varieties. It enjoys great reputation all over the world, and is cultivated in large numbers in many countries [1,2]. Kiwifruit inevitably suffers from many pests and diseases which affect the industry. The most devastating disease is kiwi canker, caused by *Pseudomonas syringae* pv. *actinidiae* (Psa) [2,3,4,5,6]. Currently, the main methods for the treatment of canker include copper preparation pesticides and biological control [7,8,9]. Although some recent chemical fungicides, such as peptides [10], neolignans [11], 1,4-benzoxazin-3-one derivatives [12], and synthetic 1,2,3-triazole-tailored carbazoles [13], have good antibacterial activity against Psa, there are increasing concerns about the harmful impacts of chemical fungicide residues on human health and the environment. Therefore, there is still a lack of safe and effective prevention methods for canker disease. Endophytic fungi, due to the process of co-evolution with the host plants, produce a series of active secondary metabolites. Our strategy is to find anti-Psa agents from kiwi-associated fungi. According to a lot of screening work, one of the kiwi-associated fungi, *Zopfiella* sp, exhibits good inhibitory activity on Psa, and chemical investigations on this fungus have been carried out. Previously, bisabolane sesquiterpenes, α-pyrone derivatives, and 3-decalinoyltetramic acid derivatives have been isolated from this fungus [14,15], while the 3-decalinoyltetramic acid derivatives such as zofielliamides A, B, and D showed anti-Psa activity with MIC values of 64, 32, and 64 μg/mL, respectively [15]. In this study, four cytochalasins, namely zopfiellasins A–D (**1**–**4**), are characterized from *Zopfiella* sp. (Figure 1). The structures with absolute configurations have been established by means of spectroscopic methods as well as single-crystal X-ray diffraction. All compounds were evaluated for their antibacterial activity against Psa. Herein, the isolation, structure elucidation, and antibacterial activity of these compounds are described.

## 2. Results and Discussion

### 2.1. Structural Identification of Compounds **1**–**4**

**Figure 2:**
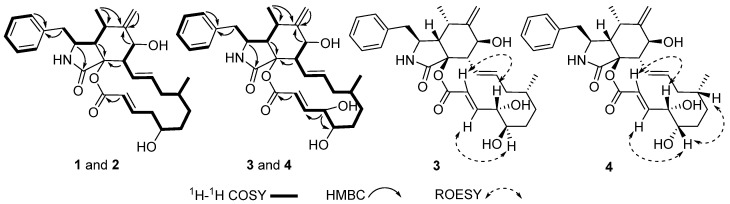
Key 2D-NMR correlations for compounds **1**–**4**.

Compound **1** was isolated as colorless crystals, while compound **2** was isolated as white powder. They were identified as a pair of epimers whose molecular formulae were established to be C_29_H_37_NO_5_ on the basis of HRESIMS data, corresponding to 12 degrees of unsaturation. Their ^13^C-NMR spectra (Table 1. For original spectra, please see the Supplementary Materials) showed 29 carbon resonances ascribable for 2CH_3_, 6CH_2_, 16CH, and 5 non-protonated carbons. In combination with ^1^H-NMR data, a mono-substituted phenyl, two trans double bonds, a terminal double bond, and two carbonyl carbons were readily identified. All these data suggested that **1** and **2** should be two cytochalasin derivatives [16,17,18,19,20]. Analysis of ^1^H–^1^H COSY data revealed several fragments, as shown in Figure 2. In addition, a detailed analysis of HMBC data suggested that **1** and **2** should have a similar structure to that of the known compound cytochalasin Z3 [16]. However, the configuration of C-19 in cytochalasin Z3 was not established. The single-crystal X-ray diffraction on compound **1** suggested C-19 to be *R* form, which allowed C-19 in compound **2** to be *S* form. This finding was supported by the ROESY analysis as well as the coupling constant modification of H-19 in **2**. Therefore, the structures of compounds **1** and **2** were identified and named as zopfiellasins A and B, respectively.

**Figure 3:**
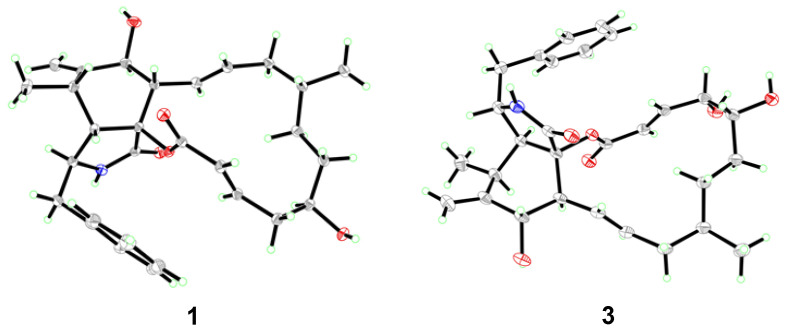
ORTEP diagrams of **1** and **3**.

Compounds **3** (colorless crystals) and **4** (white solid) were also isolated as a pair of epimers. They possessed a molecular formula C_29_H_37_NO_5_ that was established by the HRESIMS data. The 1D and 2D-NMR data of **3** and **4** were similar to those of **1** and **2** (Table 1). The MS data of **3** and **4** showed 16 mass units more than **1** and **2**, indicating that **3** and **4** should be oxidization products of **1** or **2**. Analysis of ^1^H–^1^H COSY and HMBC data indicated that **3** and **4** possessed one more hydroxy group at C-20 (Figure 2). Fortunately, the structure with the absolute configuration of **3** was determined according to single-crystal X-ray diffraction (Figure 3). In order to elucidate the stereoconfiguration of **4**, a detailed analysis of ROESY data between **3** and **4** was conducted. Although C-19 and C-20 in compounds **3** and **4** are in a 14-membered ring system, the existence of two trans double bonds makes the ring in a rigid state. Therefore, the differences in the stereoconfiguration can be detected by comparing their ROESY correlations. The ROESY data of **4** revealed almost the same patterns as those of **3**. However, the key correlation between H-19 and H-16 in **4** (Figure 2), not observed in the ROESY spectrum of **3**, suggested that C-19 in **4** should be *S* form, rather than *R* form in **3**. Therefore, compound **4** was elucidated as 19-epimer of **3**. Finally, the structures of **3** and **4** were established and named zopfiellasins C and D, respectively.

### 2.2. Antibacterial Activity against Psa

Compounds **1**–**4** were evaluated for their antibacterial activity against Psa using the previously reported method [11]. As a result, compounds **1** and **3** exhibited certain inhibitory activity on Psa with MIC values of 25 and 50 μg/mL. A brief analysis of their structure-activity relationship suggested that the stereoconfiguration of C-19 might play an important role in their antibacterial ability. The S form of C-19 in compounds **2** and **4** made them inactive. As introduced before, 3-decalinoyltetramic acid derivatives from this fungus also exhibited anti-Psa activity with MICs of 32 and 64 μg/mL. Therefore, cytochalasins and 3-decalinoyltetramic acid derivatives are suggested to be active compounds against Psa in the fungus *Zopfiella* sp. Searching for more related compounds from this fungus should be a good strategy for the discovery of anti-Psa agents.

## 3. Materials and Methods

### 3.1. Materials and Instruments

Melting points were obtained on an X-4 micro melting point apparatus (Yuhua Instrument Company, Gongyi, China). Optical rotations (OR) were recorded on a JASCO P-1020 digital polarimeter (Horiba, Kyoto, Japan). UV spectra were measured on a UH5300 UV-vis double beam spectrophotometer (Hitachi High-Technologies, Tokyo, Japan). IR spectra were carried out using a Shimadu Fourier transform infrared spectrometer with KBr pellets (Shimadu Corporation, Kyoto, Japan). NMR spectra were acquired with a Bruker Avance III 600 instrument (Bruker, Karlsruher, Germany). High-resolution electrospray ionization mass spectra (HRESIMS) were recorded on a LC-MS system consisting of a Q Exactive™ Orbitrap mass spectrometer with an ESI ion source used in an ultra-high resolution mode and a Dionex UltiMate 3000 RSLC UPLC system (ThermoFisher Scientific, Bremen, Germany). Crystallographic data were collected on a Bruker D8 QUEST diffractometer using graphite-monochromated Cu K*α* radiation. Silica gel (200–300 mesh and 500–800 mesh), RP-18 gel (40–75 µm), and Sephadex LH-20 were used for column chromatography (CC). Preparative HPLC was performed on an Agilent 1260 liquid chromatography system (Agilent Technologies, Santa Clara, CA, USA) with a Zorbax SB-C18 (5 µm, 9.4 mm × 150 mm) column and a D-detector.

### 3.2. Fungal Material and Cultivation Conditions

The fungus *Zopfiella* sp. was isolated from healthy tissue of the kiwi plant (*Actinidia chinensis* Planch). It was identified as a species of the genus *Zopfiella* by ITS sequencing with an accession number KR154941.1. Further identification of this fungus is ongoing. Culture medium consists of glucose (5%), pork peptone (0.15%), yeast (0.5%), KH_2_PO_4_ (0.05%) and MgSO_4_ (0.05%). Initial pH was adjusted to 6.0, and the fermentation was initially implemented on an Erlenmeyer flask for 6 days until the mycelium biomass reached the maximum. Following this, it was transferred to rice medium for 24 °C in dark culture for 30 days. Rice medium: 75 g of rice, 75 mL of water, placed in a 250 mL Erlenmeyer flask, sterilized at 121 °C for 15 min, a total of 200 bottles.

### 3.3. Extraction and Isolation

The rice cultural broth (15 kg) was extracted five times with EtOAc. The EtOAc layer was concentrated under reduced pressure to give an oily crude extract (200 g). The extract (200 g) was subjected to CC on silica gel (200–300 mesh) with a gradient of CHCl_3_/MeOH (1:0, 40:1, 20:1, 10:1, 5:1, 2:1, 0:1, *v*/*v*) to obtain seven fractions (A–G). Fraction B (12 g) was further separated by silica gel CC with a gradient of petroleum ether/acetone (from 10:1 to 2:1) to give eight subfractions B1–B8. Fraction B3 (600 mg) was prepared by HPLC (CH_3_CN-H_2_O from 30:70 to 60:40, *v*/*v*, 25 min) to give compound **1** (11 mg) and a mixture. The latter was purified by CC over Sephadex LH-20 (MeOH) to give compound **2** (3 mg). Fraction B6 (310 mg) was prepared by HPLC (CH_3_CN-H_2_O from 30:70 to 50:50, *v*/*v*, 25 min) to give compounds **3** (7 mg) and **4** (8 mg).

Zopfiellasin A (**1**): Colorless crystals, mp: 220–222 °C; [α]^24^_D_ + 114.5 (c 0.16, MeOH); ^1^H-NMR (600 MHz, methanol-*d*_4_) and ^13^C-NMR (150 MHz, methanol-*d*_4_) data, see Table 1; HRESIMS *m/z* 480.27461 [M + H]^+^ (calcd for C_29_H_38_NO_5_^+^: 480.27445).
Zopfiellasin B (**2**): White powder; [α]^24^_D_ + 130.1 (c 0.35, MeOH); UV (MeOH) λ_max_ (log *ε*) 215 (3.82) nm; ^1^H-NMR (600 MHz, methanol-*d*_4_) and ^13^C-NMR (150 MHz, methanol-*d*_4_) data, see Table 1; HRESIMS *m/z* 480.27446 [M + H]^+^ (calcd for C_29_H_38_NO_5_^+^: 480.27445).
Zopfiellasin C (**3**): Colorless crystals, mp: 219–220 °C; [*α*]^24^_D_ + 63.5 (c 0.80, MeOH); UV (MeOH) *λ*_max_ (log *ε*) 210 (3.76) nm; IR (KBr) *ν*_max_ 3423, 1651, 1277, 1102, 1016, 974, 703 cm^−1^; ^1^H-NMR (600 MHz, methanol-*d*_4_) and ^13^C-NMR (150 MHz, methanol-*d*_4_) data, see Table 1; HRESIMS *m/z* 496.26932 [M + H]^+^ (calcd for C_29_H_38_NO_6_^+^: 496.26936).
Zopfiellasin D (**4**): White solid; [α]^24^_D_ + 87.6 (c 0.21, MeOH); UV (MeOH) λ_max_ (log *ε*) 215 (3.82) nm; ^1^H-NMR (600 MHz, methanol-*d*_4_) and ^13^C-NMR (150 MHz, methanol-*d*_4_) data, see Table 1; HR-ESI-MS *m/z* 496.26950 [M + H]^+^ (calcd for C_29_H_38_NO_6_^+^: 496.26936).
Crystal Data for Zopfiellasin A (**1**). C_29_H_37_NO_5_, *M* = 479.59 *a* = 10.1460 (4) Å, *b* = 11.9673 (5) Å, *c* = 21.3369 (8) Å, α = 90.00°, β = 90.00°, γ = 90.00°, *V* = 2590.73 (18) Å^3^, *T* = 151 (2) K. *a* = 9.2386 (2) Å, *b* = 11.0791 (2) Å, *c* = 26.7495 (6) Å, α = 90°, β = 90°, γ = 90°, *V* = 2737.96 (10) Å^3^, *T* = 298 (2) K, space group *P*21 21 21, *Z* = 4, *μ*(Cu Kα) = 1.54178 mm^−1^. A total of 33,559 reflections were measured, of which 5559 were independent (*R_int_* = 4.22%). The final anisotropic full-matrix least-squares refinement on F^2^ with 326 variables converged at *R*_1_ = 3.02%, for the observed data and *wR*_2_ = 7.62% for all data. The goodness of fit was 1.032. The absolute configuration was determined by the Flack parameter = 0.02(4) CCDC: 2,104,459 (https://www.ccdc.cam.ac.uk).
Crystal data for Zopfiellasin C (**3**). C_29_H_37_NO_6_·CH_3_OH, *M* = 527.64, *a* = 10.4883(6) Å, *b* = 23.6229(13) Å, *c* = 11.8002(6) Å, α = 90.00°, β = 90.452(2)°, γ = 90.00°, *V* = 2923.6(3) Å^3^, *T* = 150(2) K, space group *P*1 21 1, *Z* = 4, *μ*(Cu Kα) = 0.687 mm^−1^. A total of 69,193 reflections were measured, of which 12,495 were independent (*R_int_* = 6.72%). The final anisotropic full-matrix least-squares refinement on F^2^ with 717 variables converged at *R*_1_ = 4.06%, for the observed data and w*R*_2_ = 10.97% for all data. The goodness of fit was 1.016. The absolute configuration was determined by the Flack parameter = 0.06(5). CCDC: 2,104,583 (https://www.ccdc.cam.ac.uk).

**Table 1:** ^1^H (600 MHz) and ^13^C (150 MHz)-NMR data for compounds **1**–**4** in methanol-*d*_4_.

No.	1		2		3		4	
	*δ*_C_	*δ*_H_ (*J* in Hz)	*δ*_C_	*δ*_H_ (*J* in Hz)	*δ*_C_	*δ*_H_ (*J* in Hz)	*δ*_C_	*δ*_H_ (*J* in Hz)
1	174.0, C		173.8, C		174.0, C		173.9, C	
3	54.9, CH	3.37, td (6.1, 2.7)	55.2, CH	3.34, m	54.7, CH	3.40, m	54.9, CH	3.38, m
4	48.7, CH	2.82, m	49.3, CH	2.76, dd (4.7, 3.2)	48.4, CH	2.86, d (2.8)	48.7, CH	2.82, d (5.9)
5	32.8, CH	3.17, m	33.0, CH	3.11, m	32.8, CH	3.22, m	32.9, CH	3.19, m
6	151.4, C		151.6, C		151.4, C		151.5, C	
7	71.2, CH	3.78, dd (10.9, 0.8)	70.6, CH	3.81, dd (11.3, 1.1)	71.6, CH	3.78, d (10.7)	71.2, CH	3.80, d (11.0)
8	49.6, CH	3.34, m	50.6, CH	3.18, dd (11.1, 9.9)	49.0, CH	3.38, m	49.4, CH	3.31, m
9	85.2, C		84.7, C		85.5, C		85.4, C	
10	43.9, CH_2_	2.82, m; 2.82, m	43.9, CH_2_	2.89, m; 2.89, m	43.9, CH_2_	2.78, d (5.7)	43.9, CH_2_	2.82, d (5.9)
11	14.2, CH_3_	0.85, d (6.7)	14.5, CH_3_	0.83, d (6.7)	14.1, CH_3_	0.87, d (6.7)	14.3, CH_3_	0.86, d (6.7)
12	114.3, CH_2_	5.29, s; 5.08, s	114.2, CH_2_	5.33, s; 5.09, s	114.4, CH_2_	5.27, s; 5.09, s	114.3, CH_2_	5.30, s; 5.09, s
13	128.7, CH	5.84, m	128.7, CH	5.76, m	128.8, CH	5.84, dd (15.1, 9.8)	128.9, CH	5.77, dd (15.1, 9.8)
14	136.8, CH	5.23, m	136.6, CH	5.35, m	136.6, CH	5.18, m	136.4, CH	5.24, m
15	42.9, CH_2_	2.11, m; 1.68, m	42.6, CH_2_	2.17, m; 1.75, m	43.3, CH_2_	2.09, m; 1.63, m	43.2, CH_2_	2.13, m; 1.68, m
16	35.2, CH	1.23, m	32.8, CH	1.52, m	35.1, CH	1.16, d (6.4)	33.5, CH	1.34, m
17	32.4, CH_2_	1.63, m; 0.65, m	30.7, CH_2_	1.68, m; 0.89, m	33.8, CH_2_	1.54, m; 0.62, m	30.6, CH_2_	1.62, m; 0.79, m
18	38.7, CH_2_	1.84, m; 1.19, m	34.6, CH_2_	1.69, m; 1.57, m	31.0, CH_2_	1.54, m; 1.44, m	29.7, CH_2_	1.70, m; 1.50, m
19	72.2, CH	3.59, td (9.7, 4.4)	69.7, CH	3.92, m	75.2, CH	3.59, d (9.8)	74.3, CH	3.71, dd (7.0, 6.5)
20	43.8, CH_2_	2.71, m; 2.17, m	42.7, CH_2_	2.58, m; 2.35, m	74.5, CH	4.48, s	76.0, CH	4.13, dd (6.5, 5.2)
21	148.9, CH	6.88, m	149.0, CH	7.10, m	151.4, CH	6.87, dd (15.6, 3.6)	151.0, CH	7.03, dd (15.7, 5.1)
22	123.1, CH	5.64, dd (15.6, 0.8)	123.5, CH	5.70, d (15.7)	119.6, CH	5.81, d (15.6)	121.1, CH	5.83, dd (15.7, 1.5)
23	166.0, C		166.1, C		166.2, C		166.2, C	
24	20.8, CH_3_	0.90, d (6.6)	20.5, CH_3_	0.92, d (6.6)	20.7, CH_3_	0.89, d (6.6)	20.5, CH_3_	0.89, d (6.6)
1′	138.4, C		138.8, C		138.1, C		138.3, C	
2′,6′	131.0, CH	7.13, d (7.4)	130.9, CH	7.16, d (7.4)	131.1, CH	7.13, d (7.4)	131.0, CH	7.14, d (7.4)
3′,5′	129.6, CH	7.26, dd (7.4, 7.4)	129.6, CH	7.28, dd (7.4, 7.4)	129.6, CH	7.26, dd (7.4, 7.4)	129.6, CH	7.26, dd (7.4, 7.4)
4′	127.8, CH	7.18, t (7.4)	127.8, CH	7.20, t (7.4)	127.8, CH	7.18, t (7.4)	127.9, CH	7.18, t (7.4)

### 3.4. Antibacterial Assay

The bacterium *Pseudomonas syringae* pv. *actinidiae* (Psa) was donated by Dr. He Yan of Northwest A & F University. A sample of each culture was then diluted 1000-fold in fresh L-B broth and incubated with shaking (160 rpm) at 27 °C for 10 h. The resultant mid-log phase cultures were diluted to a concentration of 5 × 10^5^ CFU/mL, and then 200 μL was added to compound-containing plates. The minimum inhibition concentration (MIC) was determined by measuring bacterial growth after 24 h by performing 1:1 serial dilutions of each compound ranging from 1–128 μg/mL. Since there is no effective antibiotic drug against Psa, no positive control was included in this experiment.

## 4. Conclusions

Four cytochalasins divided as two pairs of epimers have been isolated from a kiwi-associated fungus of the genus *Zopfiella*. Their structures with absolute configuration were determined by means of spectroscopic methods and single-crystal X-ray diffraction. Compounds **1** and **3** showed anti-Psa activity. To the best of our knowledge, this is the first report of anti-Psa activity of cytochalasin derivatives. This research makes cytochalasins a potential application prospect in agricultural antibiotics.

## Data Availability

Date of the compounds are available from the authors.

## Supplementary Materials

The following are available online. Spectra of 1D, 2D-NMR and HRESIMS for compounds **1**–**4** (PDF), X-ray crystallographic data of **1** and **3** (CIF).

## Sample Availability

Not available.
